# Mutant α-synuclein causes death of human cortical neurons via ERK1/2 and JNK activation

**DOI:** 10.1186/s13041-024-01086-6

**Published:** 2024-03-05

**Authors:** Hidefumi Suzuki, Naohiro Egawa, Keiko Imamura, Takayuki Kondo, Takako Enami, Kayoko Tsukita, Mika Suga, Yuichiro Yada, Ran Shibukawa, Ryosuke Takahashi, Haruhisa Inoue

**Affiliations:** 1 Department of Neurology, Kyoto University Graduate School of Medicine, Kyoto, Japan; 2 iPSC-Based Drug Discovery and Development Team, RIKEN BioResource Research Center (BRC), Kyoto, Japan; 3 Center for iPS Cell Research and Application (CiRA), Kyoto University, Kyoto, Japan; 4 Medical-Risk Avoidance Based on iPS Cells Team, RIKEN Center for Advanced Intelligence Project (AIP), Kyoto, Japan

**Keywords:** Synucleinopathies, *SNCA* A53T mutation, Cortical neurons, MAPK cascade, Cognitive decline

## Abstract

Synucleinopathies refer to a group of disorders characterized by SNCA/α-synuclein (α-Syn)-containing cytoplasmic inclusions and neuronal cell loss in the nervous system including the cortex, a common feature being cognitive impairment. Still, the molecular pathogenesis of cognitive decline remains poorly understood, hampering the development of effective treatments. Here, we generated induced pluripotent stem cells (iPSCs) derived from familial Parkinson’s disease (PD) patients carrying *SNCA* A53T mutation, differentiating them into cortical neurons by a direct conversion method. Patient iPSCs-derived cortical neurons harboring mutant α-Syn exhibited increased α-Syn-positive aggregates, shorter neurites, and time-dependent vulnerability. Furthermore, RNA-sequencing analysis, followed by biochemical validation, identified the activation of the ERK1/2 and JNK cascades in cortical neurons with *SNCA* A53T mutation. This result was consistent with a reverted phenotype of neuronal death in cortical neurons when treated with ERK1/2 and JNK inhibitors, respectively. Our findings emphasize the role of ERK1/2 and JNK cascades in the vulnerability of cortical neurons in synucleinopathies, and they could pave the way toward therapeutic advancements for synucleinopathies.

**Supplementary Information:**

The online version contains supplementary material available at 10.1186/s13041-024-01086-6.

## Introduction

Synucleinopathies, including Parkinson’s disease (PD) and dementia with Lewy bodies (DLB), are a heterogeneous group of neurodegenerative disorders neuropathologically defined by the abnormal accumulation of α-synuclein (α-Syn) protein and neuronal cell loss in the nervous system [1, 2]. Patients with synucleinopathies exhibit characteristic motor symptoms and a variety of non-motor deficits. Currently, there are no approved disease-modifying therapies available for these conditions.

Cognitive impairment is a major manifestation of synucleinopathies and, along with complicated motor features, plays a vital role in determining the health-related quality of life for patients [3]. Community-based studies have indicated that the majority of PD patients with long disease duration were eventually affected by dementia [4]. Neuropathological investigations have revealed that synucleinopathies with dementia have α-Syn pathology prevalent in the neocortical and limbic areas [5, 6]. Additionally, the deposition of α-Syn in neocortical regions has been found to correlate strongly with the severity of cognitive decline [7]. However, the molecular mechanisms underlying the cognitive impairment in synucleinopathies are still largely unknown.

Mutations within, and multiplications of the α-Syn-encoding *SNCA* gene cause familial forms of PD [8–10]. Moreover, the single nucleotide polymorphisms (SNPs) located in the *SNCA* gene locus have been linked with the onset of sporadic cases of synucleinopathies [11, 12]. Although the *SNCA* A53T mutation is infrequent, patients who carry this mutation display histopathological findings and clinical presentations, including dementia, similar to those seen in sporadic cases [13, 14]. This similarity points to potentially shared pathogenic mechanisms. Consequently, a detailed investigation of the *SNCA* A53T mutation model may provide deeper insights into the etiology of sporadic synucleinopathies. Since the establishment of induced pluripotent stem cell (iPSC) models derived from patients with synucleinopathies [15–19], studies of iPSC-derived midbrain dopaminergic neurons carrying *SNCA* A53T mutation have revealed an increase in nitrosative stress leading to decreased expression of peroxisome proliferator-activated receptor-γ coactivator-1α (PCG1α) [20], disrupted synaptic connectivity [21], or defective metabolic and bioenergetic processes [22]. Yet, the implications of the *SNCA* A53T mutation on cellular phenotypes and the associated pathways in cortical neurons, potentially leading to cognitive deficit, are not fully understood. In the present study, we generated cortical neurons from PD patients with *SNCA* A53T mutation by robust induction methods, with the aim of elucidating the molecular underpinnings of the cognitive decline observed in synucleinopathies.

## Results

**Fig. 1:**
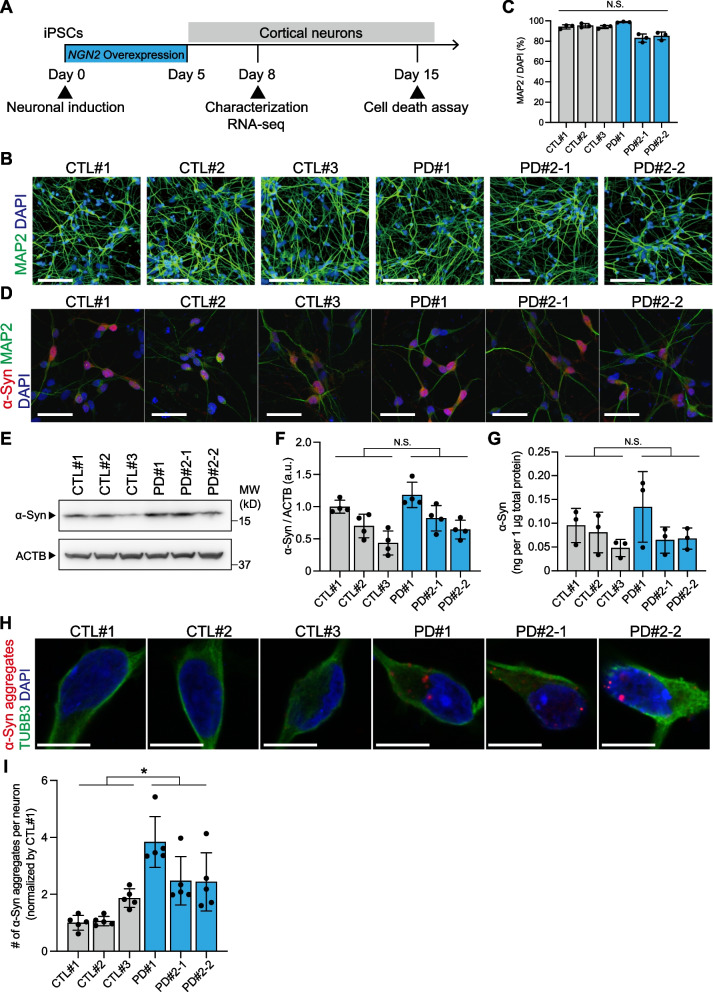
Differentiation and characterization of the cortical neurons derived from PD patients carrying *SNCA* A53T mutation. **A** Experimental timeline of differentiation and characterization of the iPSC-derived cortical neurons. Human neurogenin 2 (*NGN2*) is transiently overexpressed for 5 days for cortical neuronal induction. After induction of cortical neurons, analyses were performed from day 8 to day 15. **B** Representative images of MAP2 (green)-positive immunostaining in the differentiated neurons derived from the iPSCs on day 8 after neuronal induction. Scale bar = 100 μm. **C** Differentiation capacity of the differentiated neurons on day 8 after neuronal induction (n = 3 experimental replicates for each line; one-way ANOVA followed by Bonferroni’s multiple comparison test; *N.S.* not significant). **D** Representative images of α-synuclein (α-Syn) (red) in the differentiated neurons (green) on day 8 after neuronal induction. Scale bar = 50 μm. **E**, **F** Western blots of the differentiated neurons for α-Syn on day 8 after neuronal induction. β-actin (ACTB) was used as an internal control (n = 3 biological replicates; two-tailed Student’s *t*-test; *N.S.* not significant). **G** Quantification data of electrochemiluminescence assays of differentiated neurons for α-Syn on day 8 after neuronal induction (n = 3 biological replicates; two-tailed Student’s *t*-test; *N.S.* not significant). **H** Representative images of α-Syn-positive small aggregates (red) detected with anti-α-Syn oligomer specific antibodies in cortical neurons (green). Scale bar = 10 μm. **I** Quantification data of the number of α-Syn-positive small aggregates per neuron (n = 5 independent wells; two-tailed Student’s *t*-test; *p < 0.05). Bar graphs represent mean ± SD

To establish a cellular model for cortical neurons related to *SNCA* A53T mutation, we generated three induced pluripotent stem cell (iPSC) lines (PD#1, PD#2-1, and PD#2-2) from two patients with early-onset familial PD carrying *SNCA* A53T mutation and recruited three control iPSC lines (CTL#1, CTL#2, and CTL#3) (Additional file 1: Fig. S1A–C, Table S1). The established iPSC lines were robustly differentiated into cortical glutamatergic neurons through doxycycline (Dox)-inducible human neurogenin 2 (*NGN2*) overexpression by the previously defined direct conversion method [23] (Fig. 1A, B). Following neuronal induction for 5 days, the cultures consisted of around 90% of cortical neurons that expressed a neuronal marker, MAP2, on day 8 (Fig. 1C). There were no significant differences in differentiation capacity among the lines.

As α-Syn is a key pathogenic factor in synucleinopathies [24], we investigated whether *SNCA* A53T mutation affects its protein homeostasis and aggregation propensity in the differentiated cortical neurons. After robust induction of highly purified cortical neurons from PD patient- and control-derived iPSCs, immunocytochemistry confirmed the production of α-Syn on day 8 (Fig. 1D). There was no significant difference in *SNCA* mRNA expression levels of cortical neurons derived from PD on day 8, compared to those from the control group (Additional file 1: Fig. S2A). Similarly, the intraneuronal protein expression levels measured with western blotting and electrochemiluminescence assay showed no significant difference between PD patient- and control-derived cortical neurons on day 8 (Fig. 1E–G, Additional file 1: Fig. S2B, C). Meanwhile, we found that using anti-α-Syn oligomer-specific antibodies, α-Syn-positive small aggregates had significantly increased in the cytoplasm of the cortical neurons with *SNCA* A53T mutation compared to control-derived cortical neurons on day 8 (Fig. 1H, I, Additional file 1: Fig. S2D, E), possibly reflecting the aggregation propensity of mutant α-Syn within the cortical neurons.

**Fig. 2:**
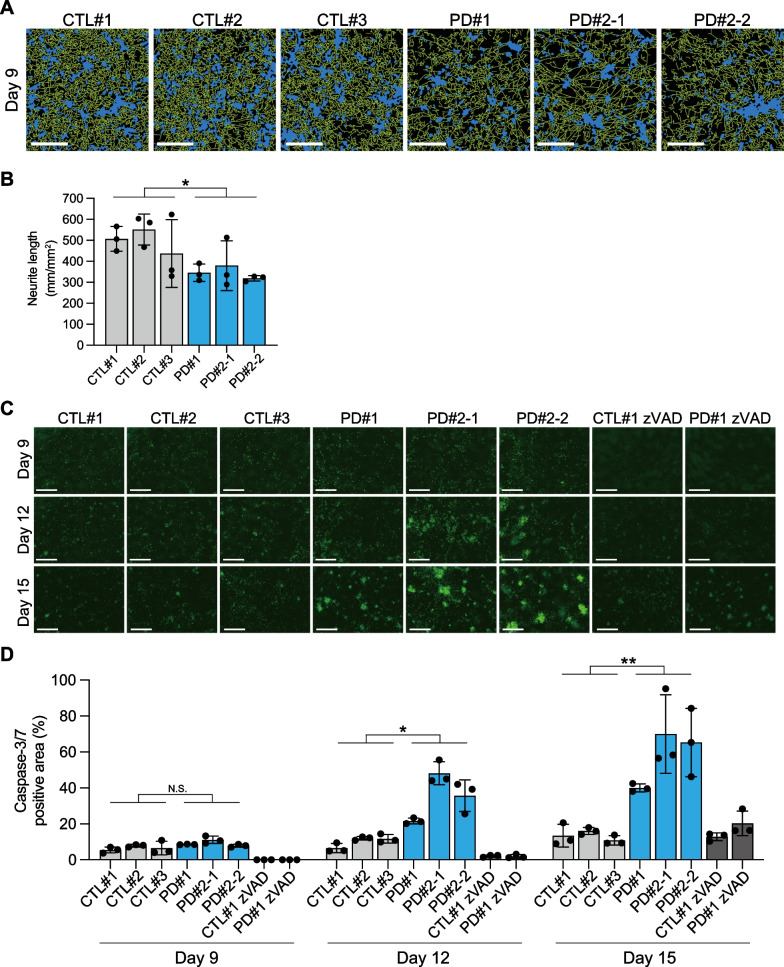
PD patient-derived cortical neurons show shorter neurite length and time-dependent vulnerability. **A** Representative images used for quantification of neurite length on day 9 after neuronal induction using the IncuCyte ZOOM imaging system. Green and blue represent identified neurites and cell body clusters, respectively. Scale bar = 200 μm. **B** Quantification data of neurite length on day 9 after neuronal induction. Neurite lengths of PD patient-derived cortical neurons are compared to control-derived cortical neurons (n = 3 biological replicates; two-tailed Student’s *t*-test; *p < 0.05). **C** Representative images of caspase-3/7 positive cells (green) on day 9, day 12, and day 15 after neuronal induction with or without Z-VAD-FMK, caspase-3/7 inhibitor. Scale bar = 400 μm. zVAD: Z-VAD-FMK. **D** Quantification data of caspase-3/7 positive cells on day 9, day 12, and day 15 after neuronal induction. PD patient-derived cortical neurons are compared to control-derived cortical neurons (n = 3 biological replicates; two-tailed Student’s *t*-test; *N.S.* not significant, *p < 0.05, **p < 0.01). Bar graphs represent mean ± SD

To investigate whether the generated cortical neurons derived from PD patients show a pathology resembling synucleinopathies, we next assessed the morphological phenotypes. To strictly align the numbers of neurons between cell lines, the generated cortical neurons were reseeded in the same number on new culture plates on day 5. We found that PD patient-derived cortical neurons exhibited reduced neurite length compared to control-derived cortical neurons (Fig. 2A, B). The average neurite length of three PD patient-derived cortical neurons was approximately 30% shorter than that of three control-derived cortical neurons on day 9, suggesting neurite elongation function would be reduced in PD patient-derived cortical neurons.

We then investigated whether PD patient-derived cortical neurons show degenerative phenotypes. We analyzed the extent of cells with activated caspase 3/7, well-known apoptosis markers, because neurodegeneration in synucleinopathies is caused by apoptosis as well as necrosis [25]. On day 8, the medium was exchanged with a new one with a reagent capable of detecting activated caspase 3/7 fluorescently. There was no difference in the ratio of activated caspase 3/7-positive cells to total cells between PD patient- and control-derived cortical neurons on day 9. However, PD patient-derived cortical neurons demonstrated a significant increase in apoptotic cells on days 12 and 15 (Fig. 2C, D). The average percentage of apoptotic cells in PD patient-derived cortical neurons was 35% and 44% higher than that of control-derived cortical neurons on days 12 and 15, respectively, suggesting gradual cell loss after neuronal maturation. Z-VAD-FMK, a pan-caspase inhibitor, reduced the percentage of apoptosis in CTL#1 and PD#1. Taken together, cortical neurons carrying *SNCA* A53T mutation exhibited shorter neurite elongation and neurodegenerative phenotype.

**Fig. 3:**
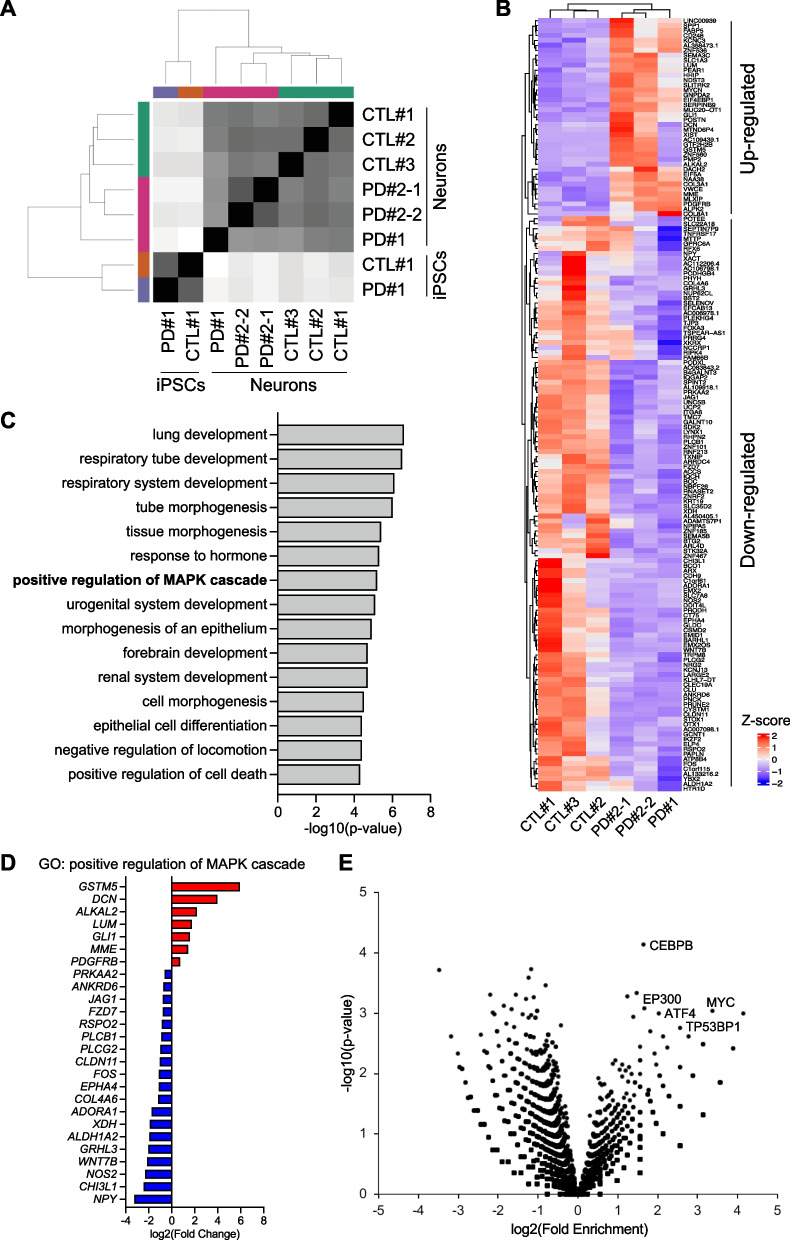
RNA-sequencing analysis implies activation of MAPK cascades in PD patient-derived cortical neurons. **A** Sample-to-sample distance heatmap of variance stabilized expression data for all six lines (CTL#1, CTL#2, CTL#3, PD#1, PD#2-1, PD#2-2) of cortical neurons on day 8 and two lines (CTL#1, PD#1) of iPSCs. The darker grey colors indicate more similar transcriptome profiles. **B** Hierarchical clustering heatmap showing differentially expressed genes (DEGs) between PD patient-derived and control-derived cortical neurons on day 8. Z-scores are used to compare expression levels between samples. Red bands and blue bands indicate higher expression and lower expression, respectively. **C** Gene ontology (GO) enrichment analysis using Metascape. Bar charts showing the top 15 GO terms among statistically significant biological processes. **D** Expression levels of the genes identified from RNA sequencing analysis that is differentially expressed and constitute the gene ontology (GO) term “positive regulation of MAPK cascade (GO:0043410)”. The x-axis represents the ratio of the average values of PD patient-derived cortical neurons to that of control-derived cortical neurons. **E** Volcano plots visualizing transcription factors that could regulate the DEGs predicted by ChIP-Atlas. Each dot represents an individual SRX ID

To further characterize PD patient-derived cortical neurons and investigate the underlying molecular mechanisms of the observed phenotypes, we next performed genome-wide transcriptome analysis. Total RNAs were extracted from all six lines (CTL#1, CTL#2, CTL#3, PD#1, PD#2-1, PD#2-2) of cortical neurons on day 8, and two lines of iPSCs (CTL#1, PD#1) as control. Clustering analysis demonstrated different gene sets of entity between PD patient and control in cortical neurons as well as iPSCs (Fig. 3A), which suggested that each transcriptome profile should depend on both the cell differentiation status and the genotype. We identified 156 differentially expressed genes (DEGs) between PD patient- and control-derived cortical neurons (False Discovery Rate < 0.1 and fold change >|1.2|), of which 40 were up-regulated and 116 were down-regulated in PD patient-derived cortical neurons (Fig. 3B, Additional file 2: Table S2). Bioinformatic analysis based on gene ontology (GO) using Metascape [26] revealed that different biological processes were perturbed in PD patient-derived cortical neurons (Fig. 3C, Additional file 3: Table S3). Given that genes that have the function of axonogenesis or cell death consist of the GO term “positive regulation of MAPK (mitogen-activated protein kinase) cascade (GO:0043410)”, this process could have an effect during neurite elongation and cell survival on day 8 (Fig. 3D). Furthermore, we examined transcription factors that could regulate differential gene expression patterns in PD patient-derived cortical neurons, and identified candidates, with CEBPB, EP300, MYC, ATF4, and TP53BP1 being the top 5 transcription factors suggested as being activated (Fig. 3E, Additional file 4: Table S4).

**Fig. 4:**
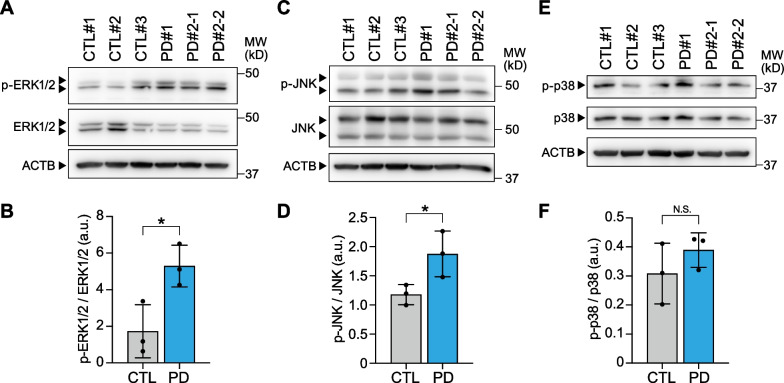
PD patient-derived cortical neurons show higher levels of phosphorylated ERK1/2 and JNK. **A**, **B** Western blots and quantification data of differentiated cortical neurons for phosphorylated ERK1/2 (p-ERK1/2), ERK1/2 and β-actin (ACTB) on day 8 after neuronal induction. Phosphorylated ERK1/2 levels were normalized by ERK1/2 levels (n = 3 biological replicates; two-tailed Student’s *t*-test; *p < 0.05). **C**, **D** Western blots and quantification data of differentiated cortical neurons for phosphorylated JNK (p-JNK), JNK and β-actin (ACTB) on day 8 after neuronal induction. Phosphorylated JNK levels were normalized by JNK levels (n = 3 biological replicates; two-tailed Student’s *t*-test; *p < 0.05). **E**, **F** Western blots and quantification data of differentiated cortical neurons for phosphorylated p38 (p-p38), p38 and β-actin (ACTB) on day 8 after neuronal induction. Phosphorylated p38 levels were normalized by p38 levels (n = 3 biological replicates; two-tailed Student’s *t*-test; *N.S.* not significant). Bar graphs represent mean ± SD

Based on the results of the transcriptome analysis, we proceeded to assess the phosphorylated protein levels of various MAPKs, including ERK1/2, JNK and p38, using western blotting to determine whether the MAPK cascades are positively regulated in PD patient-derived cortical neurons. The amounts of phosphorylated ERK1/2 and phosphorylated JNK, normalized by ERK1/2 and JNK, respectively, were shown to be significantly higher in PD patient-derived cortical neurons compared to those from control-derived cortical neurons on day 8 (Fig. 4A–D, Additional file 1: Fig. S3A–F). On the other hand, phosphorylated p38 protein levels, normalized by p38, were not significantly elevated in PD patient-derived cortical neurons (Fig. 4E, F, Additional file 1: Fig. S3G–I), suggesting that among the different MAPK cascades, the ERK1/2 and JNK cascades are activated in PD patient-derived cortical neurons.

**Fig. 5:**
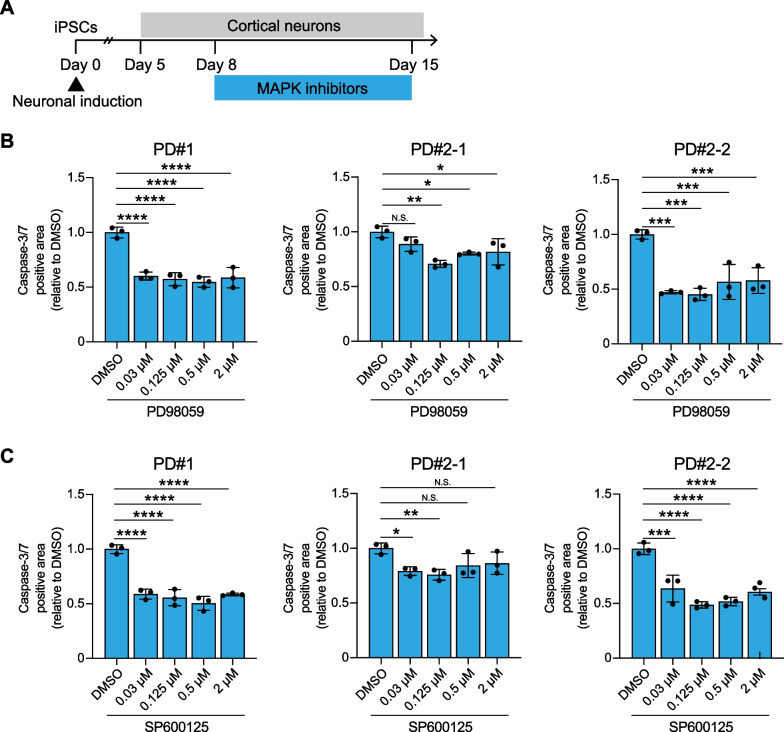
A selective ERK1/2 inhibitor and a selective JNK inhibitor restore neuronal vulnerability of PD patient-derived cortical neurons. **A** Experimental timeline of treatment with PD98059 and SP600125 for PD patient-derived cortical neurons. **B**, **C** Quantification data of caspase-3/7 positive cells on day 15 in PD#1, PD#2-1, and PD#2-2, respectively. PD98059 and SP600125 were administered separately at concentrations of 0.03 μM, 0.125 μM, 0.5 μM, and 2 μM, and DMSO was used for comparison (n = 3; one-way ANOVA followed by Dunnet’s multiple comparison test; *p < 0.05, **p < 0.01, ***p < 0.001, ****p < 0.0001, *N.S.* not significant). Bar graphs represent mean ± SD. *DMSO* dimethyl sulfoxide

To determine the involvement of the ERK1/2 and JNK cascades in the pathogenesis of PD-derived cortical neurons, we explored whether modulating these cascades would alter the pathological phenotypes. We administered a selective ERK1/2 inhibitor, PD98059, and a selective JNK inhibitor, SP600125 [27], to the neuronal culture. These inhibitors were introduced at four different concentrations, with the maximum concentration at 2 μM, on day 8 after neuronal induction, and they were allowed to incubate for the subsequent 7 days (Fig. 5A). Our results indicated that both PD98059 and SP600125 substantially reduced cell death in the cortical neurons derived from PD patients. Specifically, when compared to vehicle, PD98059 significantly suppressed the apoptotic activity in the PD#1, PD#2-1 and PD#2-2-derived cortical neurons by 45%, 29%, and 55%, respectively, at optimized concentrations (Fig. 5B). Similarly, SP600125 led to a significant reduction in apoptotic activity by 50%, 24%, and 51% in the three cell lines, respectively (Fig. 5C). Collectively, these data suggested a pivotal role of the activated ERK1/2 and JNK cascades in promoting cellular vulnerability in cortical neurons with *SNCA* A53T.

## Discussion

In the present study, we generated high-purity cortical neurons from early-onset PD patients carrying *SNCA* A53T mutation to uncover the molecular mechanisms underlying cognitive impairment seen in synucleinopathies, and the following three main findings were obtained. First, PD patient-derived cortical neurons displayed pathological phenotypes, including increased α-Syn-positive aggregates, shorter neurite elongation and time-dependent vulnerability. Second, transcriptome analysis, coupled with subsequent biochemical validation, indicated the activation of ERK1/2 and JNK cascades in PD patient-derived cortical neurons. Third, suppressing the ERK1/2 and JNK cascades mitigated cell death in PD patient-derived cortical neurons.

Cognitive dysfunction is not only a common feature of synucleinopathies but also a determinant of a patient’s health-related quality of life [3]. While understanding that the molecular basis of cognitive decline is crucial, few studies have analyzed the cellular pathologies of human-based cortical neurons. A previous study generating iPSC-derived cortical neurons with *SNCA* A53T mutation demonstrated higher nitric oxide levels, disrupted endoplasmic reticulum (ER)-associated degradation, and increased ER stress [28]. However, the study did not highlight neurodegenerative-related phenotypes or MAPK cascades. The discrepancies of cellular phenotypes between our study and previous ones can be attributed to the distinct methods used for inducing cortical neurons. For instance, the dual SMAD inhibition protocol [29] employed in a previous study produces a mixture of glial cells. In contrast, our method yields highly purified cortical neurons, enabling us to evaluate neuron-autonomous alterations.

While α-Syn aggregates were observed by immunocytochemistry, they were not detected in western blot analysis. This inconsistency can be attributed to the difficulty in detecting α-Syn aggregates in cellular models, primarily due to the reduced sensitivity in vitro [30]. Our data indicated that, while α-Syn aggregates are present and identifiable by immunocytochemistry on day 8 after neuronal induction, their accumulation at this stage is insufficient for detection by western blot analysis. Moreover, our data showed the remarkable feature of cortical neuronal vulnerability rather than α-Syn pathology. This finding may be based on a culture condition without glial cells, which increases cellular stress and contributes to accelerated neuronal death. Meanwhile, impaired neurite outgrowth and cell death have been reported in iPSC-derived dopaminergic neurons with *SNCA* multiplications and other PD-causative genes [31–33], as well as in iPSC-based amyotrophic lateral sclerosis models [34]. These similarities may suggest shared pathologies across neurodegenerative diseases rather than being exclusive to cortical neurons with *SNCA* A53T mutation.

This is the first study to reveal the involvement of the ERK1/2 and JNK activation in neuronal death associated with cortical synucleinopathies, using a human-derived disease model. While previous studies using neurotoxic animal models of PD have pointed out the activation of ERK1/2 or JNK cascades inducing neuronal cell death in synucleinopathies, they have primarily focused on nigrostriatal pathways without addressing the cortical region [35–38]. This limitation is likely due to the fact that these models are not able to replicate the pathology seen in the cortical areas. In studies using human post-mortem brain samples, aggregated phosphorylated ERK has been reported to be observed in the substantia nigra neurons of PD and DLB patients [39], but the cortical region was not examined. Regarding phosphorylated JNK, a report has indicated that there was no increase in the post-mortem brains of DLB patients [40].

ERK1/2 and JNK are activated by the phosphorylation of threonine and tyrosine residues in response to various stimuli, including oxidative stress, and transactivate specific transcriptional regulators that can induce cell apoptosis [41]. There is evidence to suggest that α-Syn binds to ERK [42]. The ERK1/2 cascade may be directly activated by aggregated α-Syn, which could lead to neuronal death. While no direct evidence currently links α-Syn to the JNK cascade, the mechanism by which mutant α-Syn activates this cascade can be postulated as follows. Studies have shown that α-Syn aggregates bind with the mitochondrial outer membrane protein TOM20, inducing impaired mitochondrial respiration and increased production of reactive oxygen species (ROS) [43]. Furthermore, α-Syn is known to activate ASK1, a MAP3K situated upstream of the JNK cascade, via oxidative stress [44]. Based on these data, it is plausible that in PD patient-derived cortical neurons, α-Syn aggregates lead to mitochondrial dysfunction. The subsequent oxidative stress could activate ASK1, thereby promoting the downstream cascade. Additionally, we speculate that the apoptosis seen in cortical neurons is executed by the following mechanisms. We predicted transcription factors responsible for the differential gene expression patterns in patient-derived cortical neurons and identified ATF4 as a candidate. ATF4 is known as a stress-induced transcription factor that promotes the induction of apoptosis [45], and it has been reported to mediate the execution of the JNK cascade [46]. Collectively, ATF4 might play a crucial role in JNK-facilitated neuronal apoptosis in cortical neurons with synucleinopathies. However, further investigations are required to determine the exact mechanism by which mutant α-Syn causes neuronal apoptosis.

Our data indicated that the ERK inhibitor PD98059 and the JNK inhibitor SP600125 can mitigate the cortical neuronal vulnerability associated with synucleinopathies. However, as these inhibitors are broad-spectrum, they may lead to potential side effects. Therefore, there are challenges that need to be addressed prior to their clinical application. This study also has the following limitations. The *SNCA* A53T mutation exhibits histopathological findings and clinical symptoms, including dementia, that are similar to those observed in sporadic synucleinopathies, suggesting potentially shared pathogenic mechanisms. However, determining whether the insights from the present study can be extended to sporadic cases or to familial synucleinopathies with different gene mutations will be crucial. In addition, we also need to consider the possibility that iPSC-derived neuronal models may not fully capture the pathophysiology of adult-onset synucleinopathies.

In conclusion, we established high-purity cortical neurons with *SNCA* A53T mutation, demonstrating phenotypes associated with synucleinopathies. Our models indicated the involvement of ERK1/2 and JNK activation in the human cortical vulnerability of synucleinopathies.

## Methods

### iPSCs generation and maintenance

We obtained somatic cells from two PD patients carrying *SNCA* A53T mutation and three healthy individuals, generating six iPSC lines in total. Among these, PD#2-1 and PD#2-2 originated from the same PD patient, allowing us to maximize the use of the available resources. iPSCs were generated and maintained under feeder-free conditions as described previously [47, 48]. Briefly, human cDNAs for reprogramming factors including OCT3/4, SOX2, KLF4, L-MYC, LIN28, and p53 carboxyl-terminal dominant-negative fragments were transduced into fibroblasts or peripheral blood mononuclear cells (PBMCs) with episomal vectors using an electroporation technique. The generated iPSCs were maintained on iMatrix-511 E8 fragment (MATRIXOME, Osaka, Japan) -coated plates in StemFit AK02N medium (Ajinomoto, Tokyo, Japan) supplemented with 1 × Penicillin–Streptomycin (Gibco, Thermo Fisher Scientific, Waltham, MA) and passaged every 7 days. 10 μM Y-27632 (Nacalai Tesque, Kyoto, Japan) was added to the medium at the time of plating and removed by replacing it with fresh medium on the next day.

### iN-iPSCs generation and conversion into cortical neurons

Tetracycline-inducible human neurogenin 2 (*NGN2*) construct was transduced into iPSCs using a *piggyBac* vector as previously described [49]. The iPSC colonies in which the construct was efficiently introduced were selected with neomycin, G418 disulfate (Nacalai Tesque). From these, subclones that could efficiently differentiate into neurons were further selected. The selected iPSCs were referred to as iN-iPSCs. For differentiation into cortical neurons, iN-iPSCs were dissociated into single cells and replated onto Matrigel (Corning Incorporated, Corning, NY)-coated plates at 300,000 cells/cm^2^ in a neuronal medium [Neurobasal plus medium (Gibco, Thermo Fisher Scientific), 1X B27 plus (Gibco, Thermo Fisher Scientific), 1X GlutaMAX (Gibco, Thermo Fisher Scientific) and 1 × Penicillin–Streptomycin (Gibco, Thermo Fisher Scientific)], supplemented with 10 μM Y-27632 (Nacalai Tesque) and 1 μg/mL doxycycline hydrochloride (Wako Pure Chemicals Industries, Osaka, Japan). On day 5, the differentiated neurons were dissociated into single cells with TrypLE select (Gibco, Thermo Fisher Scientific) and reseeded onto Matrigel-coated 96-well plates (Corning Incorporated) at 60,000 cells/cm^2^ in the neuronal medium with 10 μM Y-27632.

### Genotyping

Genomic DNA was extracted with PureLink Genomic DNA Mini Kit (Invitrogen, Thermo Fisher Scientific) and then amplified with KOD FX Neo (TOYOBO, Osaka, Japan). Specific primer pairs used for screening *SNCA* gene mutation were as follows: forward 5′-TTGTGCTAAAATCGTAATTGG-3′; reverse 5′-TTAGAATGCTCAGTGATTGTTTTAC-3′. Following purification of the amplified PCR products with QIAquick PCR Purification Kit (QIAGEN, Hilden, Germany), cycle sequencing was performed using BigDye Terminator v3.1 Cycle Sequencing Kit (Applied Biosystems, Thermo Fisher Scientific). The sequencing reactions were purified with BigDye XTerminator Purification Kit (Applied Biosystems, Thermo Fisher Scientific), and the 3500 xL Genetic Analyzer (Applied Biosystems, Thermo Fisher Scientific) was used for capillary electrophoresis. Sequencing data were visualized with SnapGene software (GSL Biotech LLC).

### Immunocytochemistry

Cells were fixed with 4% paraformaldehyde (PFA) (Nacalai Tesque) in 1X phosphate-buffered saline (PBS) (Nacalai Tesque) for 15 min at room temperature. The fixed cells were washed with PBS, permeabilized with 0.1% Triton X-100 (Nacalai Tesque) in PBS for 10 min, and incubated with Blocking One Histo (Nacalai Tesque) for 30 min at room temperature. Subsequently, the cells were incubated with primary antibodies overnight at 4 °C. Primary antibodies used in this study were as follows: chicken anti-MAP2 (1:5000, Cat#ab5392, RRID:AB_2138153, Abcam, Cambridge, MA), rabbit anti-α-synuclein (MJFR1, 1:2000, Cat#ab138501, RRID:AB_2537217, Abcam), mouse anti-human alpha-synuclein (ASyO5, 1:1000, Cat#AS13 2718, RRID:AB_2629502, AgriSera, Vannas, Sweden), mouse anti-TRA-1-60 (1:1000, Cat#4746, RRID:AB_2119059, Cell Signaling Technology, Danvers, MA), mouse anti-Stage-Specific Embryonic Antigen-4 (SSEA-4) (1:1000, Cat#MAB4304, RRID:AB_177629, Millipore, Burlington, MA), rabbit anti-NANOG (1:500, Cat#RCAB004P-F, RRID:AB_1560380, ReproCELL Incorporated, Yokohama, Japan), mouse anti-βIII tubulin (1:1000, Cat#MAB1637, RRID:AB_2210524, Millipore), rabbit anti-βIII tubulin (1:2000, Cat#ab18207, RRID:AB_444319, Abcam), goat anti-SOX17 (1:1000, Cat#AF1924, RRID:AB_355060, R&D Systems, Minneapolis, MN), mouse anti-αSMA (1:500, Cat#M0851, RRID:AB_2313736, DAKO, Glostrup, Denmark). The cells were incubated with Alexa Fluor-conjugated secondary antibodies (1:1000, Invitrogen, Thermo Fisher Scientific) for 1 h at room temperature. All antibodies were diluted in PBS-T containing 5% Blocking One Histo. DAPI (Invitrogen, Thermo Fisher Scientific) was used for nuclei counterstaining. Fluorescence images were acquired using the following fluorescent microscopes: BZ-X710 (KEYENCE, Osaka, Japan), OperaPhenix (PerkinElmer, Waltham, MA), InCell analyzer 6500 (Cytiva, Tokyo, Japan), and FV3000 (Olympus, Tokyo, Japan).

### qPCR

Total RNA was isolated using miRNeasy Mini Kit (QIAGEN). Complementary DNA (cDNA) was synthesized using ReverTra Ace qPCR RT kit (TOYOBO) with 1 μg of total RNA. Real-time PCR was performed with SYBR Premix Ex Taq II (Takara Bio Inc., Shiga, Japan) using QuantStudio 12 K Flex Real-Time PCR System (Applied Biosystems, Thermo Fisher Scientific). Primer pairs used for this study were as follows: *SNCA* forward 5′-GGAGTGGCCATTCGACGAC-3′; *SNCA* reverse 5′-CCTGCTGCTTCTGCCACAC-3′, *GAPDH* forward 5′-GTCTCCTCTGACTTCAACAGCG-3′; *GAPDH* reverse 5′-ACCACCCTGTTGCTGTAGCCAA-3′. The expression level of the *SNCA* gene was normalized to corresponding *GAPDH* values.

### Western blotting

Cell lysates were prepared with a radioimmunoprecipitation assay (RIPA) buffer (Nacalai Tesque) containing protease and phosphatase inhibitor cocktails (Roche, Basel, Switzerland). Samples were centrifuged at 20,000×*g* for 30 min at 4 ℃. Total protein concentrations of the supernatants were quantified using the bicinchoninic acid (BCA) assay (Pierce, Thermo Fisher Scientific). 10–30 μg total protein was mixed with 6 × sample buffer solution (Nacalai Tesque) containing dithiothreitol (Nacalai Tesque) and separated in a 10–20% SDS–polyacrylamide gel electrophoresis (PAGE) gels, and then transferred onto polyvinylidene fluoride (PVDF) membrane (Millipore). For α-synuclein detection, membranes were treated with 0.4% (w/v) PFA in PBS for 30 min at room temperature before blocking with PVDF blocking reagent for Can Get Signal (TOYOBO) for 1 h at room temperature. Membranes were incubated with primary antibodies against rabbit anti-α-synuclein (MJFR1, 1:1000, Cat#ab138501, RRID:AB_2537217, Abcam) and rabbit anti-phospho-ERK1/2 (1:2000, Cat#4370, RRID:AB_2315112, Cell Signaling Technology), rabbit anti-phospho-JNK (1:1000, Cat#9251, RRID:AB_331659, Cell Signaling Technology), rabbit anti-phospho-p38 (1:1000, Cat#9211, RRID:AB_331641, Cell Signaling Technology), mouse anti-ERK1/2 (1:2000, Cat#4696, RRID:AB_390780, Cell Signaling Technology), rabbit anti-JNK (1:1000, Cat#9252, RRID:AB_2250373, Cell Signaling Technology), rabbit anti-p38 (1:1000, Cat#9212, RRID:AB_330713, Cell Signaling Technology), and mouse anti-β-actin (1:5000, Cat#A5441, RRID:AB_476744, Sigma-Aldrich, St. Louis, MO) overnight at 4℃. Membranes were incubated with horseradish peroxidase-conjugated secondary antibodies (GE HealthCare, Chicago, Il) for 1 h at room temperature and visualized using an ECL Prime detection kit (GE HealthCare) and ImageQuant LAS 4000 (GE HealthCare). Signal intensities were normalized to those of β-actin.

### Electrochemiluminescence assay

Preparation of cell lysates and quantification of total protein concentration were performed using the same procedure as for western blot analysis. α-synuclein protein concentrations were measured with U-PLEX Human α-synuclein Kit (Meso Scale Discovery, Rockville, MD) and MESO SECTOR S 600 (Meso Scale Discovery) as per the manufacturer’s instructions. The biotinylated capture antibody and SULFO-TAG-conjugated detection antibody that we used in the assay were those packaged in the kit. To compare among conditions, quantified α-synuclein concentrations were normalized by total protein concentrations.

### Neurite length measurement and cell death assay

Cortical neurons were generated as described earlier. On day 8, the medium was totally removed and replaced with neuronal medium containing 5 μM CellEvent Caspase-3/7 Green Detection Reagent (Invitrogen, Thermo Fisher Scientific). Depending on the experiments, 5 μM z-VAD-FMK (Peptide Institute Inc., Osaka, Japan), 0.1% dimethyl sulfoxide (DMSO) (Sigma-Aldrich), or different concentrations of PD98059 (ChemScene, Monmouth Junction, NJ) and SP600125 (ChemScene) were added to the medium. After replacing the medium, the plates were incubated in IncuCyte S3 (Sartorius, Goettingen, Germany) and imaged using the IncuCyte ZOOM imaging system (Essen BioScience, Sartorius) with images collected every 6 h from day 8 to day 15. During this period no medium exchange was performed. We defined neurite length per cell-body cluster area (mm/mm^2^) as neurite length (mm/mm^2^), and areas showing green fluorescence per total area (%) as caspase-3/7 positive area (%) according to the manufacturer’s instructions.

### RNA sequencing analysis

On day 8, two iPSC lines (CTL#1 and PD#1) and six cortical neurons were used for RNA sequence analysis. Total RNA was isolated using miRNeasy Mini Kit (QIAGEN). After analysis of the RNA integrity number (RIN) and concentration using 4200 TapeStation System (Agilent Technologies, Santa Clara, CA), library construction was performed with KAPA mRNA HyperPrep Kit (Kapa Biosystems, Cape Town, South Africa) as per the manufacturer’s instructions. Following quantification with the KAPA Library Quantification Kit (Kapa Biosystems), library samples were sequenced on NextSeq 550 (Illumina, San Diego, CA) using 75 bp paired-end sequencing. The quality of the raw RNA sequencing data was checked with TrimGalore (https://github.com/FelixKrueger/TrimGalore/issues/25), and then the sequencing reads were mapped against the human reference genome, GRCh38/hg38, using STAR (https://github.com/alexdobin/STAR) and RSeQC [50]. Expression for each gene in all samples was counted using featureCounts (version 1.6.3) [51]. Clustering and differential gene expression analysis were implemented with R (version 4.3.1) and DESeq2 packages (version 1.40.2). Go enrichment analysis was conducted using Metascape (version 3.5) [26]. ChIP-Atlas [52] was used to predict transcription factors that regulate the genes differentially expressed.

### Statistical analysis

Results were statistically analyzed using a two-tailed Student’s *t*-test for comparisons of two groups, and a one-way ANOVA followed by Bonferroni’s test or Dunnet’s test for multiple comparisons. p < 0.05 were considered significant. All analyses were performed using GraphPad Prism (version 9.5.0) (GraphPad Software, San Diego, CA).

### Supplementary Information

**Additional file 1: Figure S1.** Characterization of the iPSCs. **A** Representative images of iPSCs showing embryonic stem cells (ESCs)-like morphology (phase images) and expression of the pluripotent stem cell markers TRA-1-60, SSEA4, NANOG. Three iPSC lines were established from healthy individuals (CTL#, CTL#2, and CTL#3) and Parkinson’s disease (PD) patients carrying *SNCA *A53T mutation (PD#1, PD#2-1, and PD#2-2), respectively. Scale bar = 100 μm. **B** DNA sequencing analysis illustrating a heterozygous mutation (c.G209A), which resulted in p.A53T mutation in the *SNCA *gene of the iPSCs derived from the PD patients. **C** Representative images of *in vitro *embryoid body formation assay showing the expression of an ectoderm marker (TUBB3), an endoderm marker (SOX17), and a mesoderm marker (αSMA). Scale bar = 100 μm.** Figure S2.** Characterization of the cortical neurons related to α-Syn. **A** *SNCA *mRNA expression analyzed by real-time qPCR on day 8 after neuronal induction (n = 3 biological replicates; two-tailed Student’s *t*-test; *N.S.* not significant). **B**, **C** Full lengths of western blot images for Figure 1C. **D** Representative low-magnification images obtained with anti-α-Syn oligomer specific antibodies in cortical neurons. Scale bar = 200 μm. **E** Orthogonal view of α-Syn-positive small aggregates (red) detected with anti-α-Syn oligomer specific antibodies in PD#1-derived cortical neurons (green). Scale bar = 20 μm. **Figure S3.** Full-length western blot images related to MAPK cascades. **A**–**C** Full-length blot images for Fig. 4A. **D**–**F** Full length blot images for Fig. 4C. **G**–**I** Full length blot images for Fig. 4E. **Table S1.** List of the iPSC lines.

**Additional file 2: Table S2.** List of genes differentially expressed between PD patient- and control-derived cortical neurons.

**Additional file 3: Table S3.** List of Gene Ontology (GO) biological process terms identified through enrichment analysis.

**Additional file 4: Table S4.** List of transcription factors (TFs) predicted to regulate differential gene expression patterns in cortical neurons derived from PD patients.

## Data Availability

The datasets used and/or analyzed during the current study available from the corresponding author on reasonable request.
