# Supplementary material for: Mutant α-synuclein causes death of human cortical neurons via ERK1/2 and JNK activation
Source: Mol Brain. 2024 Mar 5;17:14. doi: 10.1186/s13041-024-01086-6 (PMC10916047; doi:10.1186/s13041-024-01086-6)
Supplement: Supplementary file 1 — Additional file 1: Figure S1. Characterization of the iPSCs. A Representative images of iPSCs showing embryonic stem cells (ESCs)-like morphology (phase images) and expression of the pluripotent stem cell markers TRA-1-60, SSEA4, NANOG. Three iPSC lines were established from healthy individuals (CTL#, CTL#2, and CTL#3) and Parkinson’s disease (PD) patients carrying SNCA A53T mutation (PD#1, PD#2-1, and PD#2-2), respectively. Scale bar = 100 μm. B DNA sequencing analysis illustrating a heterozygous mutation (c.G209A), which resulted in p.A53T mutation in the SNCA gene of the iPSCs derived from the PD patients. C Representative images of in vitro embryoid body formation assay showing the expression of an ectoderm marker (TUBB3), an endoderm marker (SOX17), and a mesoderm marker (αSMA). Scale bar = 100 μm. Figure S2. Characterization of the cortical neurons related to α-Syn. A SNCA mRNA expression analyzed by real-time qPCR on day 8 after neuronal induction (n = 3 biological replicates; two-tailed Student’s t-test; N.S. not significant). B, C Full lengths of western blot images for Figure 1C. D Representative low-magnification images obtained with anti-α-Syn oligomer specific antibodies in cortical neurons. Scale bar = 200 μm. E Orthogonal view of α-Syn-positive small aggregates (red) detected with anti-α-Syn oligomer specific antibodies in PD#1-derived cortical neurons (green). Scale bar = 20 μm. Figure S3. Full-length western blot images related to MAPK cascades. A–C Full-length blot images for Fig. 4A. D–F Full length blot images for Fig. 4C. G–I Full length blot images for Fig. 4E. Table S1. List of the iPSC lines. [file 13041_2024_1086_MOESM1_ESM.pdf]

Additional file 1

Title of manuscript:
Mutant α-synuclein causes death of human cortical neurons via ERK1/2 and JNK activation

Authors:
Hidefumi Suzuki, Naohiro Egawa, Keiko Imamura, Takayuki Kondo, Takako Enami, Kayoko Tsukita, Mika Suga, Yuichiro Yada, Ran Shibukawa, Ryosuke Takahashi, Haruhisa Inoue

Figure S1. Characterization of the iPSCs.
Figure S2. Characterization of the cortical neurons related to α-Syn.
Figure S3. Full-length western blot images related to MAPK cascades.
Table S1. List of the iPSC lines.

Figure S1

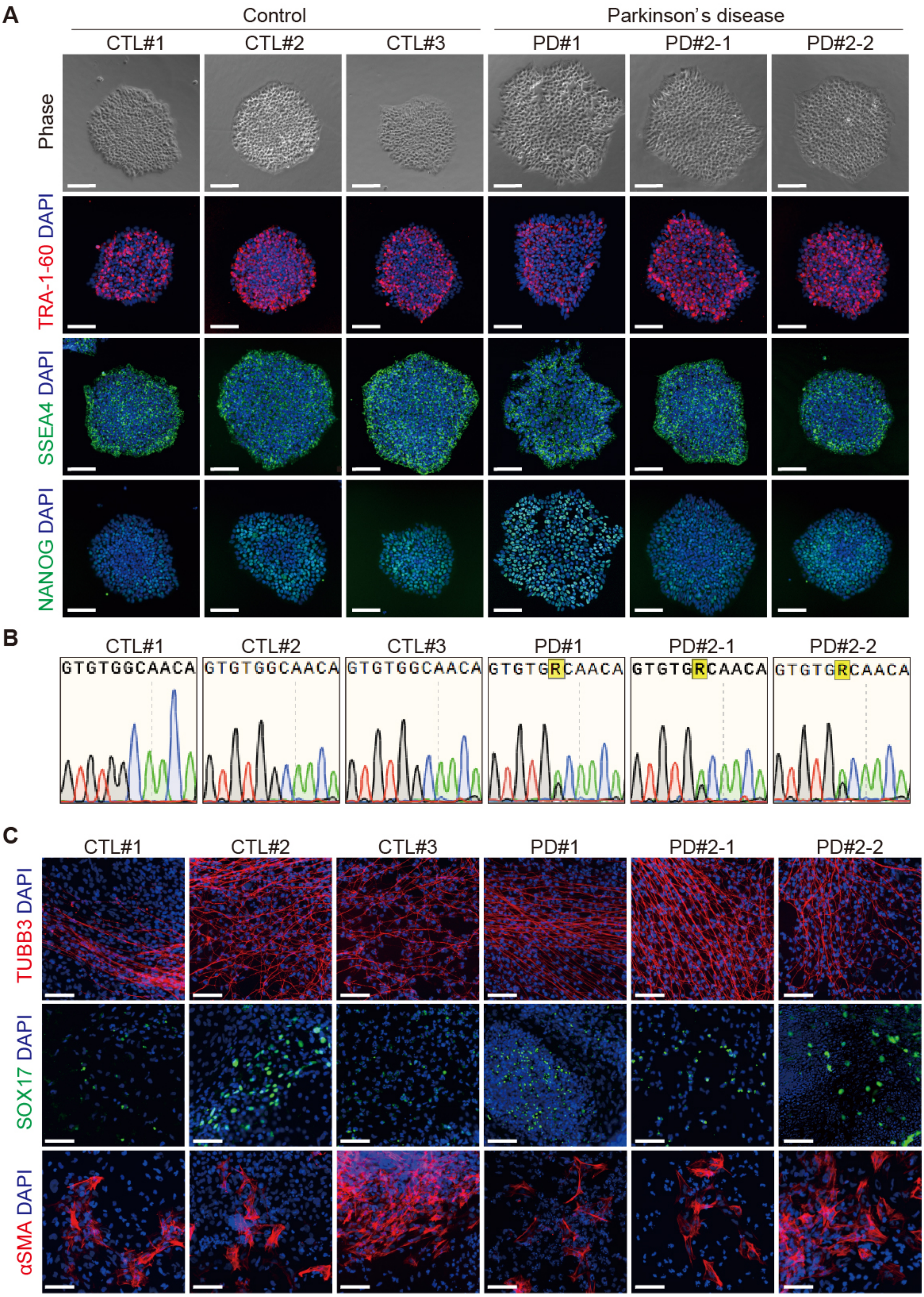
A
Control
Parkinson's disease
CTL#1
CTL#2
CTL#3
PD#1
PD#2-1
PD#2-2
Phase
TRA-1-60 DAPI
SSEA4 DAPI
NANOG DAPI
B
CTL#1
CTL#2
CTL#3
PD#1
PD#2-1
PD#2-2
GTGTGGCAACA
GTGTGGCAACA
GTGTGGCAACA
GTGTGRCAACA
GTGTGRCAACA
GTGTGRCAACA
C
CTL#1
CTL#2
CTL#3
PD#1
PD#2-1
PD#2-2
TUBB3 DAPI
SOX17 DAPI
αSMA DAPI

Figure S1. Characterization of the iPSCs.

(A) Representative images of iPSCs showing embryonic stem cells (ESCs)-like morphology (phase images) and expression of the pluripotent stem cell markers TRA-1-60, SSEA4, NANOG. Three iPSC lines were established from healthy individuals (CTL#, CTL#2, and CTL#3) and Parkinson's disease (PD) patients carrying *SNCA* A53T mutation (PD#1, PD#2-1, and PD#2-2), respectively. Scale bar = 100 μm. (B) DNA sequencing analysis illustrating a heterozygous mutation (c.G209A), which resulted in p.A53T mutation in the *SNCA* gene of the iPSCs derived from the PD patients. (C) Representative images of *in vitro* embryoid body formation assay showing the expression of an ectoderm marker (TUBB3), an endoderm marker (SOX17), and a mesoderm marker (αSMA). Scale bar = 100 μm.

Figure S2

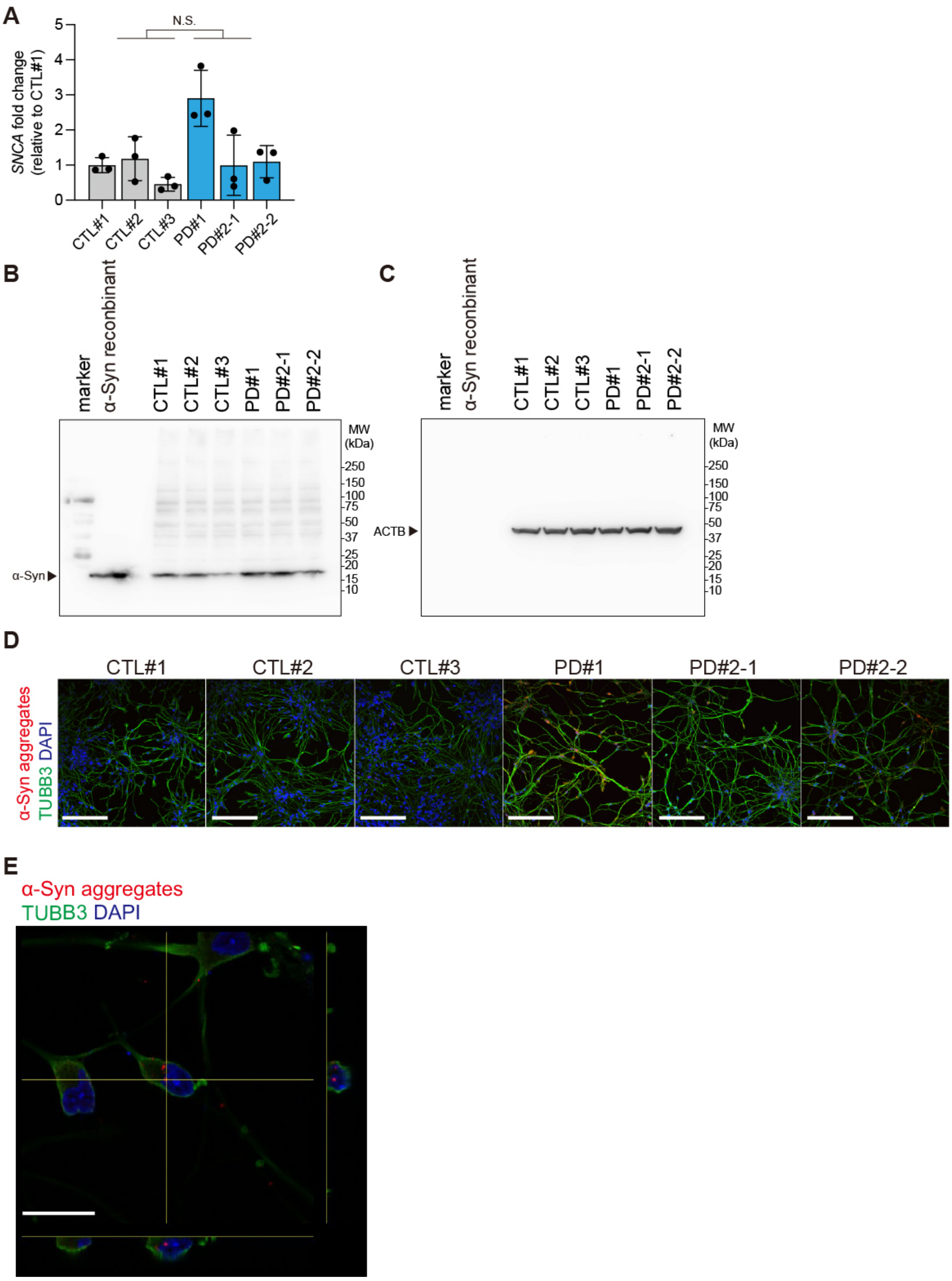

Figure S2. Characterization of the cortical neurons related to α-Syn.
(A) *SNCA* mRNA expression analyzed by real-time qPCR on day 8 after neuronal induction (n = 3 biological replicates; two-tailed Student's *t*-test; N.S.: not significant). (B, C) Full lengths of western blot images for Figure 1C. (D) Representative low-magnification images obtained with anti-α-Syn oligomer specific antibodies in cortical neurons. Scale bar = 200 µm. (E) Orthogonal view of α-Syn-positive small aggregates (red) detected with anti-α-Syn oligomer specific antibodies in PD#1-derived cortical neurons (green). Scale bar = 20 µm.

Figure S3

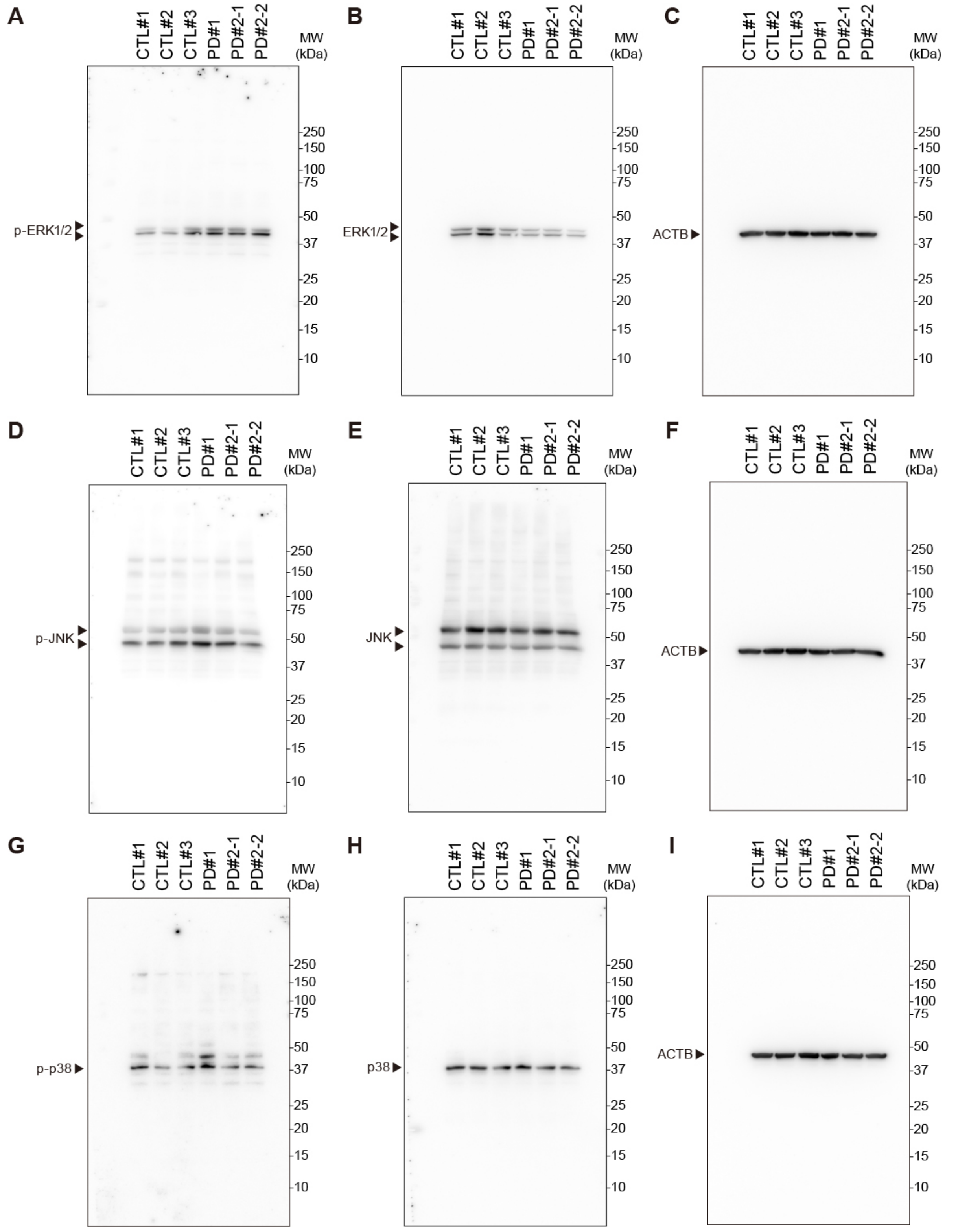

Figure S3. Full-length western blot images related to MAPK cascades.

(A-C) Full-length blot images for Figure 4A. (D-F) Full length blot images for Figure 4C. (G-I) Full length blot images for Figure 4E.

Table S1. List of the iPSC lines.

Clone name	CTL#1	CTL#2	CTL#3	PD#1	PD#2-1	PD#2-2
Age	61	65	39	38	51	51
Sex	male	female	female	male	female	female
Clinical status	healthy	healthy	healthy	familial PD	familial PD	familial PD
Genotype	wild-type	wild-type	wild-type	*SNCA* A53T	*SNCA* A53T	*SNCA* A53T
Somatic cell	PBMC	HDF	HDF	PBMC	HDF	HDF

Abbreviations: CTL, control; PD, Parkinson's disease; PBMC, peripheral blood mononuclear cell; HDF, human dermal fibroblast
